# Predictors of facility-based delivery utilization in central Ethiopia: A case-control study

**DOI:** 10.1371/journal.pone.0261360

**Published:** 2022-01-21

**Authors:** Ayalneh Demissie, Alemayehu Worku, Yemane Berhane

**Affiliations:** 1 Arsi University, College of Health Sciences, Arsi, Ethiopia; 2 Addis Ababa University, School of Public Health, Addis Ababa, Ethiopia; 3 Department of Epidemiology and Biostatistics, Addis Continental Institute of Public Health, Addis Ababa, Ethiopia; 4 Department of Reproductive Health and Population, Addis Continental Institute of Public Health, Addis Ababa, Ethiopia; Federal University of Sergipe, BRAZIL

## Abstract

**Background:**

Improving access to maternal health services has been a priority for the health sector in low-income countries; the utilization of facility delivery services has remained low. Although Ethiopia provides free maternal health services in all public health facilities utilization of services has not been as expected.

**Objective:**

This study examined predictors of facility delivery service utilization in central Ethiopia.

**Methods:**

We conducted a community-based case-control study within the catchment areas of selected public health facilities in central Ethiopia. Women who delivered their last child in a health facility were considered as cases and women who delivered their last child at home were considered as controls. Data were collected using a structured questionnaire. Multivariable logistic regression analysis was used to identify independent predictors of facility delivery utilization.

**Result:**

Facility delivery was positive and strongly associated with practicing birth preparedness and complication readiness (BPCR) (AOR = 12.3, 95%CI: 3.9, 39.1); partners’ involvement about obstetric assistance (AOR = 3.1, 95%CI: 1.0, 9.0); spending 30 or less minutes to decide on the place of delivery and 45 or less minutes to walk to health facilities (AOR = 7.4, 95%CI: 2.4, 23.2 and AOR = 8.1, 95%CI: 2.5, 26.9, respectively). Additionally, having knowledge of obstetric complication, attending ≥ 4 antenatal care (ANC) visits, birth order and the use of free ambulance service also showed significant association with facility delivery.

**Conclusion:**

Despite the availability of free maternal services there are still many barriers to utilization of delivery services. Strengthening efforts to bring delivery services closer to home and enhancing BPCR are necessary to increase institutional delivery service utilization.

## Introduction

Efforts to end preventable maternal and newborn deaths in low income settings have been hampered by low utilization of maternal health services [1]. Promotion of Facility-based delivery (FBD) is one of the vital interventions in the reduction of maternal mortality. Improved access to and high coverage of institutional delivery alone could prevent about 50% of maternal deaths [2]. Yet, almost 75% of women in Ethiopia deliver at home, a practical barrier in the reduction of maternal and neonatal mortality [3]. In Sub-Saharan Africa (SSA) and Ethiopia utilization of facility-based delivery was affected by individual attributes (educational status (both), women’s autonomy to make decisions on birth places, cultural and traditional beliefs, and wealth status) [4–8], obstetric attributes (parity and ANC use) [9] and barriers related to availability and accessibility of health services (unavailability and cost of transportation, distance to facility and limited knowledge about the benefits of institutional delivery [10–13]. Whereas: better education, higher socioeconomic status, urban residence, lower birth order, ANC visits, availability of transport, having good knowledge of obstetric complications, knowledge of BPCR, partners’ involvement to seek obstetric assistance, and women’s participation in social networks are the most identified facilitators associated with facility delivery utilization [14–21].

Although there are many factors that influence the use of health facility for delivery, the economic barrier was strong; the cost of transportation and service fees is high for the majority of women in low income countries [22]. That has prompted many African countries to introduce a free delivery services [23,24]. Since 2013, the Government of Ethiopia has put in place a policy to provide free delivery services in all public health facilities coupled with free ambulance services to facilitate referral when necessary [25].

However, utilization of institutional delivery services in Ethiopia has remained very low at 26% and 19% in Oromia region (local setting). Vast studies in Africa also showed mixed effects; some showed positive effects [26], others showed free service provision not predicating utilization of facility-based delivery [27,28], and others showed effects only in certain population groups heterogeneously [29]. Nevertheless, perception of facility delivery beyond the economic barrier is shaped by culture, personal experience and by the social discourse of risk. Social engagement can provide information on facility, details of the health care system, and can change the demand for services by affecting the perceived merit of the available services. Culture related child birth beliefs strongly influence the decision to seek care rather than her recent knowledge of the appropriate action to take. For example, in the western corner of the country, 71% of Gumuz women gave birth alone in the forest (reasons: due to fear the child could die and fear of gods curse not to destroy the family [30].

Although Ethiopia introduced free health facility delivery services in 2013, which was one of the main barriers to utilization of delivery services and extensive published studies describing the utilization of facility-based delivery in the country, to our knowledge predictors of maternal health service utilization have not been previously studied in the context of free service provision in Ethiopia. Not taking the removal of user fees into account leads to biased estimates of facility-based delivery utilization and to underestimation of financial barrier. Predicating utilization of facility-based delivery in the context of free service provision adds to the growing body of evidence on the utilization of facility-based delivery and its barriers. More robust knowledge about these factors is crucial to inform user fees policy research. Findings may be used as an advocacy tool for policymakers and health managers to develop policies and strategies that could lead to increases women’s access to and use of health facilities for delivery. Also, the findings have the potential of promising social change in the lives of women.

This study fills the gaps in the literature by predicating predictors of free maternal health service utilizations in Ethiopia. The study attempts to highlight the importance of other demand-side factors such as sharing detailed information about the health care system and services provided. Literature on the use of health services proposes that user fees are not the only factors that determine access to care. Social discourse of risk factors also plays an important role in enabling the utilization of free facility-based deliveries. Thus, this study tries to predict the contribution of: individual, obstetric and program related factors on the utilization of facility-based delivery.

## Methods

### Ethical approval

Ethical approval was obtained from Arsi University Ethical Review Committee (ERC 0122/2012) and supportive letter was obtained from East-Shewa zone Health Bureau. To get access to the community a written request sent to the local leaders and we obtained written informed consent from all participated mothers prior to their participation with informing the purpose, benefits, confidentiality of the information, and voluntary nature of participation in the study. Name and other personal identifiers were not recorded to maintain confidentiality.

### Study setting

The study was conducted in East-Shewa zone of Oromia regional state in central Ethiopia. Health services in the study area were provided through 13 primary health care facilities at the time of data collection, which comprise health posts; health centers and district hospitals. The administration as part of a nationwide initiative began implementing free delivery services in 2013 in all public health centers. Health posts located in the villages are the first point of contact with the Ethiopian health care system and is staffed by the Health Extension Workers (HEWs) who provide basic health promotion and prevention services [31]. The HEWs also keeps a family folder where they register births regardless of the place of delivery by regularly visiting households. At the community level, women encouraged to be organized into voluntary groups; to increase women’s access to and use Maternal, Newborn and Child Health services. They are called Health Development Army (HDA) and also referred to as the Women’s Development Groups (WDGs). It is a network of women in rural areas, organized into development groups of 30 women (1–30 networks), who are further clustered into groups of 6 (1–5 networks). Groups select a leader who is then trained and supervised by the HEW. The HDA leaders help members adopt practices promoted by the HEW, hold participatory learning and action meetings, link pregnant women with care providers, hold monthly meetings for pregnant women, mobilize communities to contribute resources to make facilities mother friendly and facilitate the use of either traditional or modern ambulances. It is community based social movement and major breakthrough where the government takes the lead to address maternal and child health issues. Its detail described elsewhere previously. The health centers serve as the first referral unit for health posts and provide basic emergency obstetric care (EmOC) services. The primary (district) hospitals serve as the next referral level and provide obstetric surgical interventions. All are interconnected through a referral system and a free ambulance service is available for obstetrical emergencies. The free ambulance service may not be always available and mothers may need to find their own transport for referral. There were 13 health centers at the time of data collection for this study; of which five were randomly selected for the study. The study was conducted in communities within the catchment population of the selected health centers.

### Study design

A community-based case-control study was conducted in two urban and three rural districts in central Ethiopia, to identify predictors associated with utilization of facility delivery services. The cases comprised of women who delivered their last child in a health facility in the 5 years preceding the survey. The controls included women who delivered their last child at home during the reference period.

### Sample size and sampling procedure

The sample size was calculated using OpenEpi sample size calculator [32] with the following assumptions: 95% confidence, 80% power, proportion of exposure controls to the Knowledge of obstetric complication as 62.9%, odds ratio of 2.36, a case to control ratio of 1:1 and a 4% contingency for non-responses. This gave a total sample size of 125 cases and 125 controls. This study tries to reveal the contribution of demand side factor (social discourse /public awareness) that lead to access the available free services, so we used knowledge of obstetric complication as the exposure variable for calculating the sample size.

Women of childbearing age (15–49) who gave birth in the preceding 5 years of this survey were eligible for the study. Women who delivered the index child (the most recent delivery) in a health facility were considered as cases and women who delivered the index child at home were considered as controls. Women were excluded if they are not resident in the health center catchment area for at least 6 months. Cases and controls were randomly selected from the HEWs record which was used as a sampling frame. Controls were matched to cases by the residence and closeness of births within ± 2 months. Simple random sampling was used to select the required number of cases and controls separately using the health post record as a sampling frame, proportional to population sizes of the respective health center’s catchment population. To control for potential intra household correlation, one woman was selected randomly by the lottery method if there are two or more eligible women per household. If mothers gave more than one birth within the last five years only the recent was considered for this study.

### Data collection

Data were collected using a questionnaire adapted from the demographic and health survey tools and other relevant literature. The questionnaire was initially developed in English and then translated into Afan Oromo (the regional state language) and back translated, the English version maternal Health Questionnaire is available in the supporting information as **S1 File**. The questionnaire was designed to capture three categories of determinants including socio-demographic, obstetric, and program related. The field work was conducted from June-July 2019.

Women were interviewed at home by trained data collectors who were fluent in Afan Oromo following sensitivity analysis. Each interview was carried out face-to-face where the data collector blind about the status of participants either to the case or a control group and lasted approximately 50 minutes.

### Measures

Utilization of facility delivery service for the index child was the outcome of the study. The predictors considered in the study included socio-demographic and obstetric characteristics, and a number of programmatic variables. We examined the predictive value of a number of socio-demographic variables included women’s age, education and occupation, women’s autonomy −having the ability to decide either by herself or with her husband where to deliver her last child, or else it is considered as non-autonomous, husband’s education and occupation, and socio-economic status. Then we examined respondents’ obstetric characteristics: ANC use during their recent pregnancy and the number of visits, age at first pregnancy, gravidity, parity and delivery service use. Lastly the study assessed programmatic variables: availability of health information, obstetric knowledge, availability of monthly health conference to women, aware of prior arrangements for birth, decision time for birth place (> 30 minutes / ≤30 minutes), travel time (> 45minutes / ≤ 45 minutes), availability of transport, and mother’s BPCR knowledge and practice. Knowledge of obstetrics was assessed by constructing composite scores of eighteen different items on danger signs of pregnancy, labour & delivery (LD) and postpartum (PP). The final scores categorized into “Adequate", "Fair" and “Inadequate "scales. Respondents with score 75% and above were categorize as having "Adequate knowledge"; those scored between 50 to 74% as having "fair knowledge"; and those scored less than 50% as having " Inadequate knowledge". Partner’s role towards birth planning was measured by eight questions using a five-point Likert scale. A mean score was calculated from the total response and those who scored the mean and above were categorized as partners’ involvement permissive while those scored below the mean as non-permissive. The study used to construct a summary indicator of BPCR arrangement suggested by the JHPIEGO BPCR monitoring tool [33]. Based on the six arrangements (identified place of delivery or not, identified skilled birth attendant or not, saved money or not, identified means of emergency transport or not, arranged a blood donor for emergency or not, and identified health institution with 24 h EmOC or not). The resulting BPCR index was used to examine the levels of BPCR practice among women. Women were categorized into not well prepared (less than average preparation) and well prepared (more than average preparation /prepared > = 4 of the BPCR activities).

### Data processing and analysis

The completed questionnaires were checked for their completeness and consistency, then double-entered, cleaned and analyzed using Stata version 12. To investigate whether there was a recall bias, a sensitivity analysis was conducted using only those women that gave birth in the previous 6 months to assess potential for recall bias and asked six questions. The questions included age at first pregnancy, gravidity, parity, history of abortion, history of stillbirth and income. The analysis did not alter the findings, and thus, all participants were retained. Categorical and continuous variables were summarized as proportions and means respectively. Cross-tabulations comparing cases versus controls were performed. Chi-square (Fischer’s exact test was used to see the association between groups where statistical significance defined as alpha less than 0.05 (two-sided).

A conditional binary logistic regression (which takes into account the matching design) was used to determine the effect of independent variables on facility delivery.

The crude regression model was adjusted for known confounders, including the marital status, religion, women’s autonomy, occupation, education, and income, age at first pregnancy, history of stillbirth, parity, ANC visit, gestational age, travel and decision time, mode of transport, and partners’ role. The confounders were selected a priori based on the literature on drivers and deterrents of facility delivery in sub-Saharan Africa. Then variables whose p-value was < 0.25 in bivariate analysis and aprior selected key variables were fitted into a multivariate logistic regression model. The Hosmer-Lemeshow test was used to check model fitness. Then, a p-value < 0.05 in multivariate logistic regression was considered as statistically significant and the strength of associations was determined using the adjusted odds ratio (AOR) with the corresponding 95% confidence interval, the raw data set used in this analysis is available in the supporting information as **S2 File [34]**.

## Results

### Socio-demographic characteristics of the study participants

A total of 125 cases and 122 controls participated in this study that leads to a response rate of 98%—. Cases that do not have controls as the pairs were excluded. The mean age of the cases was 27 (SD = 5 years) which was similar to the mean age of controls of 27 (SD = 6 years, P>0.05). The vast majority (65%) of both cases and controls were below the age of 30 years. A similar proportion of cases and controls (94% and 98%, respectively) were married or lived as married. A higher proportion of cases and their spouses, (61% and 63%) compared to controls and their spouses (23% and 39%), respectively had primary and above education (P<0.05). Over 70% of both the cases and controls were housewives. However, 29% cases compared with 20% controls had some private business (P>0.05). Family income significantly associated with facility delivery. Around half of cases 47% compared to controls (16%) had monthly income 2501 ETB or more (P <0.001). A higher proportion of cases (61%) compared to controls (43%) made a self decision to attend a health facility (P <0.01). As shown in Table 1, on a bivariable analysis, there was a significant difference between cases and controls in their educational status, household expenditure and women’s autonomy (Table 1).

**Table 1:** Socio-demographic characteristics and women’s autonomy of study participants’ by place of delivery central Ethiopia, 2021.

Variables		Facility delivery		Home delivery		χ2* square, d_f_ and p- value
		n	%	n	%	
**Age of respondent**	15–24	37	30	38	31	χ^2^2.17 = df = 3 P-value > 0.05
	25–29	38	31	42	34	
	30–34	31	25	22	18	
	35+	16	13	20	16	
	Total	122	100	122	100	
**Mean age (SD)**		27(5)		27(6)		
**Marital status**	Married	115	94	120	98	χ^2^2.88 = df = 1 P-value > 0.05
	Single	7	6	2	2	
	Total	122	100	122	100	
**Religion**	Orthodox	85	70	76	62	χ^2^2.13 = df = 3 P-value > 0.05
	Moslem	16	13	24	20	
	Protestant	18	15	19	16	
	Traditional	3	2	3	2	
	Total	122	100	122	100	
**Occupation**	Housewife	85	70	95	78	χ^2^3.99 = df = 3 P-value > 0.05
	Employee	11	9	11	9	
	Private business	25	20	14	11	
	Other, specify	1	1	2	2	
	Total	122	100	122	100	
**Women’s education**	Illiterate	29	24	72	59	χ^2^40.15 = df = 2 P-value < 0.001
	Read & write	18	15	22	18	
	Primary & above	75	61	28	23	
**Husband’s education**	Illiterate	24	21	47	39	χ^2^20.55 = df = 3 P-value < 0.001
	Read & write	19	17	26	22	
	Primary education	49	43	42	35	
	Secondary & above	23	20	5	4	
**Monthly income in Birr**	< = 2500ETB	65	53	102	84	χ^2^25.98 = df = 1 P-value < 0.001
	2501+ ETB	57	47	20	16	
	Total	122	100	122	100	
**Mean monthly family Income Birr**		2510ETB		1830ETB		
**Women’s autonomy**	Non autonomous	47	39	70	57	χ^2^8.69 = df = 1 P-value < 0.01
	Autonomous	75	61	52	43	
	Total	122	100	122	100	

Note:

* χ2 test and df: degree of freedom.

### Obstetric characteristics of the study participants

Nearly one third 31% of the cases compared to 15% of controls were primipara mothers, and 16% cases and 33% controls were grand multiparous (parity >4). A higher proportion of controls (66%) compared to (48%) of cases had their first pregnancy before the age of 20. Over 80% of both the cases and controls attended ANC. Although ANC attendance was high, 82% of controls and 43% of cases had had less than four antenatal visits. A third of the controls 33% and 11% of cases who attended ANC booked in the third trimester. A higher proportion of cases (59%) compared to controls (26%) spent 30 or few minutes to decide on the place of delivery. The majority of cases (75%) spent 45 or less minutes to walk to the nearest health facility compared to a small proportion of the controls (30%) (P <0.001) and only a small proportion of the controls (40%) used transport in contrast to 77% of cases (P <0.001). On a bivariable analysis shown in Table 2, there was a significant difference between cases and controls in terms of parity, number of ANC visits; decision and travel time, and use of transport services (**Table 2**).

**Table 2:** Obstetric service use and characteristics of the study participants by place of delivery in central Ethiopia, 2021.

Variables		Facility delivery		Home delivery		χ2 square, d_f_ and p- value
		n	%	n	%	
**Age at first marriage**	< 20 Years	79	65	101	83	χ^2^10.25 = df = 1 P-value < 0.01
	20 + Years	43	35	21	17	
**Age at first pregnancy**	< 20 Years	59	48	81	66	χ^2^8.11 = df = 1 P-value < 0.05
	20 + Years	63	52	41	34	
**Number of live births**	1	106	87	111	91	χ^2^2.42 = df = 1 P-value > 0.05
	≥ 2	16	13	11	9	
**Number of abortion**	0	57	47	44	36	χ^2^1.66 = df = 1 P-value > 0.05
	≥ 1	64	53	78	64	
**Number of still birth**	0	115	94	112	92	χ^2^0.57 = df = 1 P-value > 0.05
	≥ 1	7	6	10	8	
**Gravidity**	1	38	31	18	15	χ^2^13.81 = df = 2 P-value < 0.01
	2–3	64	52	64	52	
	4+	20	16	40	33	
**Parity**	1 birth	52	43	41	34	χ^2^ 9.29 = df = 2 P-value < 0.05
	2–3 births	55	45	47	39	
	4+ births	15	12	34	28	
**ANC in the very latest pregnancy**	No	15	12	21	17	χ^2^1.17 = df = 1 P-value > 0.05
	Yes	107	88	101	83	
**Gestational age at first ANC visit**	≤ 12 weeks	36	30	24	24	χ^2^2.95 = df = 2 P-value > 0.05
	12–24 weeks	63	53	52	51	
	25+weeks	20	17	26	25	
**Number of ANC visits**	1–3 visits	51	43	84	82	χ^2^34.69 = df = 1 P-value < 0.001
	≥ 4 visits	68	57	19	18	
**Decision time**	< = 30 minutes	72	59	32	26	χ^2^26.81 = df = 1 P-value < .001
	>30 minutes	50	41	90	74	
**Travel time**	< = 45 minutes	92	75	37	30	χ^2^49.75 = df = 1 P-value < .001
	>45 minutes	30	25	85	70	
**Transportation to HF**	Foot	28	23	73	60	χ^2^34.21 = df = 1 P-value < .001
	Transport	94	77	49	40	
	Total	122	100	122	100	

Note: * χ2 test and df: degree of freedom.

### Knowledge and practice of birth preparedness

#### BPCR knowledge and performance

The majority of cases (79%) and only a small proportion of controls (23%) knew that they should make arrangements for the birth of a child (P <0.001). Likewise, a higher proportion of cases (68%) compared to controls (28%) had adequate BPCR knowledge (P <0.001). Similarly, a higher proportion of cases (53%) compared to controls (37%) involved their partners’ in seeking obstetric assistance (P <0.05) see (**Table 3**).

**Table 3:** BPCR knowledge and performance of study participants by place of delivery in central Ethiopia, 2021.

Variables		Facility delivery		Home delivery		χ2 square, d_f_ and p- value
		n	%	n	%	
**Knowledge of pregnancy related problems**	Inadequate	41	34	73	60	χ^2^ 16.86 = df = 1 P-value < 0.001
	Adequate	81	66	49	40	
**Knowledge of LD complications**	Inadequate	43	35	67	55	χ^2^ 9.54 = df = 1 P-value < 0.01
	Adequate	79	65	55	45	
**Knowledge of PP complications**	Inadequate	68	56	101	83	χ^2^ 20.96 = df = 1 P-value < 0.001
	Adequate	54	44	21	17	
**General knowledge of obstetric complications**	Inadequate	39	32	76	62	χ^2^ 22.52 = df = 1 P-value < 0.001
	Adequate	83	68	46	38	
**Knowledge of advantage of LD services**	Inadequate	14	11	43	35	χ^2^ 19.25 = df = 1 P-value < 0.001
	Adequate	108	89	79	65	
**Community mobilization participation**	No	91	75	104	85	χ^2^4.03 = df = 1 P-value < 0.05
	Yes	31	25	18	15	
**BPCR advises**	Yes	111	92	73	60	χ^2^32.09 = df = 1 P-value < 0.001
	No	10	8	49	40	
**Any arrangements for birth**	No	26	21	94	77	χ^2^75.82 = df = 1 P-value < 0.001
	Yes	96	79	28	23	
**Knowledge of BPCR**	Inadequate	39	32	76	62	χ^2^ 22.52 = df = 1 P-value < 0.001
	Adequate	83	68	46	38	
**BPCR status** **Prepared (> = 4 arrangements made)** **Not Prepared (<4 arrangements made)**	Not prepared	14	12	46	38	χ^2^21.04 = df = 1 P-value < 0.001
	Prepared	103	88	76	62	
**Partners’ involvement**	Non-permissive	57	47	77	63	χ^2^ 6.62 = df = 1 P-value < 0.05
	Permissive	65	53	45	37	

Note: * χ2 test and df: degree of freedom.

### Predictors of facility delivery service utilization

As shown in Table 4, the multivariable analysis results indicate significant association with facility delivery included: mothers who knew about any of the BPCR arrangement/s 12 times more likely to deliver at a health facility than the one who didn’t (AOR = 12.3, 95%CI: 3.9, 39.1), as were those mothers who had adequate knowledge about BPCR was 4 times more likely to deliver in a health facility than their counterpart. (AOR = 4.7, 95%CI: 1.3, 16.6). Mothers who claimed partners’ involvement to seek obstetric assistance were 3 times more likely to deliver at a health facility than their counterpart (AOR = 3.0, 95% CI: 1.0, 9.0). Time to decide (on the place of delivery) and travel (to the nearest health facility) appeared to be significantly associated with deliveries. Over one-third of the cases (59%) spent 30 or few minutes to decide on the place of delivery than controls (26%) (AOR = 7.4, 95% CI: 2.4, 23.2) and more cases spent 45 or less minutes to walk to the nearest health facility (75%) than controls (30%) (AOR = 8.1, 95% CI: 2.5, 26.9) (Table 4).

**Table 4:** Bivariable and multivariable analysis of predictors significantly associated with facility delivery in central Ethiopia, 2021.

Variables		Facility delivery	Home delivery	COR(95%CI)	AOR(95%CI)
**Women’s education**	Illiterate	29(24%)	72 (59%)	1	1
	Read & write	18(15%)	22(18%)	2.03(0.95, 4.33)*	1.16(0.21, 6.44)
	Primary & above	75(61%)	28(23%)	6.65 (3.61, 12.26)***	2.10 (0.38, 11.53)
**Husband’s education**	Illiterate	24(21%)	47(39%)	1	1
	Read & write	19(17%)	26(22%)	1.43 (0.66, 3.09)	1.20 (0.24, 6.15)
	Primary education	49(43%)	42(35%)	2.28 (1.20, 4.34)*	1.19 (0.27, 5.37)
	Secondary & above	23(20%)	5(4%)	9.01 (3.04, 26.66)***	1.36 (0.11, 17.23)
**Monthly income**	< = 2500ETB	65(53%)	102 (84%)	1	1
	2501+ ETB	57(47%)	20(16%)	6.47 (2.46, 8.12)***	1.96 (0.57, 6.79)
**Travel time**	>45 minutes	30(25%)	85(70%)	1	1
	< = 45minutes	92(75%)	37(30%)	7.05 (4.01, 12.39)***	8.13 (2.45, 26.95)**
**Women’s autonomy**	Non autonomous	47(39%)	70(57%)	1	1
	Autonomous	75(61%)	52(43%)	2.15 (1.29, 3.58)**	1.93 (0.67, 5.54)
**Decision time**	>30 minutes	50(41%)	90(74%)	1	1
	< = 30 minutes	72(59%)	32(26%)	4.05 (2.36, 6.96)***	7.42 (2.37, 23.24)**
**Transport use**	Foot	28(23%)	73(60%)	1	1
	Vehicle	94(77%)	49(40%)	5.00 (2.87, 8.72)***	4.92 (1.65, 14.69)**
**Age at first pregnancy**	< 20 Years	59(48%)	81(66%)	0.47 (0.28, 0.79)*	0.67 (0.24, 2.01)
	20 + Years	63(52%)	41(34%)	1	1
**Parity**	1 birth	52(43%)	41(34%)	2.88 (1.38, 5.98)**	0.59 (0.13,2 .74)
	2–3 births	55(45%)	47(39%)	2.65 (1.29, 5.46)**	0.16 (0.03, 0.77)*
	4+ births	15(12%)	34(28%)	1	1
**Number of ANC visit**	1–3 visits	51(43%)	84(82%)	1	1
	> = 4 visits	68(57%)	19(18%)	5.89 (3.18, 10.92)***	3.25 (1.05, 10.05)*
**Community mobilization participation**	N0	91(75%)	104(85%)	1	1
	Yes	31(25%)	18(15%)	1.97 (1.03, 3.75)*	1.36 (0.37, 4.99)
**Any arrangements for birth**	No	26(21%)	94(77%)	1	1
	Yes	96(79%)	28(23%)	12.39 (6.77, 22.69)***	12.29 (3.87, 39.09)***
**BPACR status** **Prepared (> = 4 arrangements made)** **Not Prepared (<4 arrangements made)**	Not prepared	14(12%)	46(38%)	1	1
	Prepared	103(88%)	76(62%)	4.45 (2.28, 8.68)***	2.32 (0.47, 11.48)
**knowledge of LD complications**	Inadequate	43(35%)	67(55%)	1	1
	Adequate	79(65%)	55(45%)	2.24 (1.34, 3.75)**	0.71 (0.22, 2.28)
**knowledge of advantage of LD services**	Inadequate	14(11%)	43(35%)	1	1
	Adequate	108(89%)	79(65%)	4.19 (2.15, 8.20)***	0.58 (0.14, 2.45)
**Knowledge of BPCR**	Inadequate	39(32%)	76(62%)	1	1
	Adequate	83(68%)	46(38%)	6.39 (3.49, 11.70)***	4.72 (1.34, 16.56)*
**Partners’ involvement**	Non**-**permissive	57(47%)	77(63%)	1	1
	Permissive	65(53%)	45(37%)	1.95 (1.17, 3.25)*	3.01 (1.01, 9.03)*

**Note:** *** very highly significant at p < 0.001

** highly significant at p < 0.01

* significant at p < 0.05.

## Discussion

This study shows distance to a health facility and birth preparedness to be the strongest predictors of utilization of institutional delivery services. These, along with other predictors are longstanding barriers to increasing uptake of institutional delivery services in many low-income settings.

In this study, women who knew any of the prior arrangements were found to be more likely to utilize facility delivery services as compared to those women who didn’t. This result is consistent with earlier studies in Ethiopia [35,36] and in other SSA countries [37].The possible explanation could be the increases in the level of knowledge about complication readiness and the recognition of danger signs and anticipation of problems can motivate mothers and their families to make arrangements for facility delivery.

Mothers who claimed to involve their partners’ in seeking obstetric assistance were more likely to deliver at the health facility compared to those mothers who didn’t involve. In patriarchal societies where women’s autonomy is undermined, making such decisions together with partners can play a significant role in improving uptake of institutional deliverer services. That can also facilitate access to family financial resources and to obtain other logistical support for facility delivery. The involvement of family members also facilitates obtaining assistance with household chores while the mother is away for facility delivery [38], otherwise men in low-income settings may typically expect women to stay at home and attend to household chores [39]. Even when there is no treatment cost, facility delivery may incur transportation and other costs that are not covered by health facilities including purchasing consumables needed for the provision of delivery services [40–43].

The study found those mothers who spent 45 or less minutes to walk to the nearest health facility were more likely to deliver at the facility. Women living closer to health facilities perceive the investment in time and money to reach care as reasonable. Long distance travels compromise women’s family responsibility and are expensive due to transport costs. Several studies elsewhere have stressed the importance of easy access to health facilities that provide delivery services [44–46] and it is also consistent with other studies previously conducted in Ethiopia [47–51]. To overcome the barriers related to travel time and shortage of transportation in rural areas, Ethiopia introduced a free ambulance service at the district level [52].

Availability of free ambulance to a HF increases the likelihood of utilizing facility delivery services in areas where the ambulance services are well organized, similar interventions were successfully implemented in Kenya and South Africa [53,54]. Household income level is an important proxy for decision making power of families for utilizing health services. Poorer families are less likely to afford transportation and additional health facilities costs and thus unlikely to have a facility delivery, despite being free. However, household income was not significantly associated with facility delivery utilization in this study inconsistent to other studies conducted in LMICs. Studies in other regions of Ethiopia also showed that women with a better economic level were more likely to utilize facility delivery services [55,56], unlike our finding. The reason might be this study tries to elucidate utilization of facility delivery in the context of free user fees for maternity services as well as ambulance for women to deliver. Mothers who visited health facilities for ANC 4 or more times during their recent pregnancy were more like to deliver in health facility than those who didn’t. This result was consistent with previous studies conducted in Assosa and Dembecha districts and Ghana [57–59]. The possible explanation might be due to the reason that ANC provides opportunities for mothers to get counseled about BPCR that can promote mothers to deliver at health facility with skilled attendants.

This study has several strengths but also some limitations. The strength includes it being one of the first studies in Ethiopia in the context of free maternal health services. The limitations include recall of some exposures, especially among multiparous women, but it is likely to be similar in cases and controls. Although we tried to control for many potential confounders, it is possible that residual confounding could still remain. However, the predictors identified are very strong with large odds ratio and unlikely to be affected by residual confounders. We have not also considered quality and cultural acceptability of facility delivery in this study. We recommend a qualitative study to understand better the predictors of facility delivery in specific contexts.

### Conclusions

Provision of free maternal initiative alone is not sufficient to increase utilization of delivery services to the desired level. Efforts must be intensified to remove other barriers to utilization of delivery services in low income settings. Further efforts to overcome limitation related to distance to health facilities and improve awareness about birth preparedness of mothers.

## Supporting information

S1 File Maternal health questionnaire. (DOCX)

S2 File Raw data set, case control. (XLSX)
