# Supplementary material for: Predictors of facility-based delivery utilization in central Ethiopia: A case-control study
Source: PLoS One. 2022 Jan 21;17(1):e0261360. doi: 10.1371/journal.pone.0261360 (PMC8782499; doi:10.1371/journal.pone.0261360)
Supplement: S1 File — (DOCX) [file pone.0261360.s001.docx]

**Maternal Health Survey Questionnaire—Women Arsi University College of Health Sciences**

| **Questionnaire No.** |  |
| --- | --- |
| **Household ID No.** |  |

**Time Interview Started: Hour: _____ Minute: _____**

**Time Interview Ended: Hour: _____ Minute: _____**

| **Name of Head of Household** |  |
| --- | --- |
| **Location** | **Urban---------------------1 Rural-----------------------2** |

| **Interviewer visits** | | | |
| --- | --- | --- | --- |
|  | **1** | **2** | **3** |
| **Date** |  |  |  |
| **Result*** |  |  |  |
| **Next visit: date** |  |  |  |
| **Time** |  |  |  |

*** Result codes:**

| **1 = Completed**  **2 = Household absent** | **3 = Incomplete interview**  **4 = Refused** | **5 = Other: (specify): ______________** |
| --- | --- | --- |

**Verbal Consent Form**

Good morning/afternoon/evening. My name is _________________________________.

(Interviewer)

I represent [NAME OF GROUP]. We are speaking with women and their families about the experience

of being pregnant and of having children. The results of this survey will be used to help improve health programs for women.

You have been selected for the interview by means of a random or chance selection process, much like picking an orange out of a basket without looking. I would like to ask you a few questions if I may, but you can refuse to answer any question I ask. You may end the interview at any time. You can also refuse to participate in the study entirely. The interview will last approximately [TIME ESTIMATE]. The information we collect from you will not be shown to anyone outside of this project.

May I proceed with the questions? Yes/No

**____________________________ __________________**

Name of interviewer Date

**Section 1. Respondent’s Socio demographic information**

| First, I would like to ask you some questions about yourself and the pregnancies you’ve had | | | | | |
| --- | --- | --- | --- | --- | --- |
| ***No*** | ***Questions*** | ***Alternative /choice of response*** | | | ***Skip*** |
| 101 | Age in Years at interview time | | ______________ | < 15/> 49 stop | |
| 102 | Marital Status | Married/in union------ 1 single ----- 2 Divorced----- 3 Widowed-------- 4 Other,Specify____________97 | | |  |
| 103 | Have you ever been pregnant in the past? | Yes ............................................................ 1  No.............................................................. 2 | | |  |
| 104 | Are you pregnant now? | Yes ............................................................ 1  No.............................................................. 2 | | |  |
| 105 | Religion | Orthodox--------1 Moslem ------ 2 Protestant-----3  Catholic-------------4 Other,Specify____________97 | | |  |
| 106 | Have you given birth in the last 24 months, either to a baby that was born alive or a baby that was born dead? | Yes ............................................................ 1  No.............................................................. 2 | | |  |
| 107 | Ethnicity | Oromo ----- 1 Amahara ----- 2  Guragie----- 3 Tigre ----- 4  Others [Specify]__________97 | | |  |
| 108 | Occupation | Housewife----------1 Employee--------------2  Farmer------------3 Private business---------4  Others,Specify___________________________97 | | |  |
| 109 | Educational Status | Illiterate-------------1 Read and write----2  primary Education (1- 8)----------3  Secondary education (9-10/12)-----4  Technical/vocational and above (10+)---5 | | |  |
| 110 | Husband’s Education Status | Illiterate-------------1 Read and write----2  primary Education (1- 8)----------3  Secondary education (9-10/12)-----4  Technical/vocational and above (10+)---5 | | |  |
| 111 | Do you have monthly income | Yes--------1 No---------2 I don’t want to tell--------3 | | | 2&3 → Sec. 2 |
|  | Monthly Income in Birr | ­­­­­­­­­­­­________________ | | |  |

**Section 2. Obstetric Factors**

| ***No*** | ***Questions*** | ***Alternative /choice of response*** | | ***Skip*** |
| --- | --- | --- | --- | --- |
| 201 | Age at first marriage | ______________ | |  |
| 202 | Age at first Pregnancy | ______________ | |  |
| 203 | Gravidity(Total No. of pregnancy) | _______ | 1.Number of Pregnancies: _____ |  |
|  |  |  | 2.Number of abortions : _____ |  |
| 204 | Parity(Total No. of births) | ______ | 1.Number of children alive : _____ |  |
|  |  |  | 2.Number of children died: _____ |  |
|  |  |  | 3.Number of still birth : _____ |  |

**Section 3. Knowledge**

| ***No*** | ***Questions*** | ***Alternative /choice of response*** | | | | ***Skip*** |
| --- | --- | --- | --- | --- | --- | --- |
| 301 | Are there some health problems that can occur during pregnancy that could endanger the life of a pregnant woman? | Yes—1 No------2 Don't know--------98 | | | |  |
| 302 | Can you mention obstetric problems that can occur during pregnancy?  (Probe: ask for the problems which are not mentioned spontaneously) | Vaginal bleeding-------------------------------------01  Severe headache-------------------------------------02  High fever ---------------------------------------------03  Swollen feet/face --------------------- 04  Loss of consciousness----------------------------- 05  Continuous vomiting ----------------------------- 06  Others,Specify___________________________97  Don’t know ...........................................98 | | | | DK→304 |
| 303 | Are there some obstetric problems that can occur during labor and child birth that could endanger the life of a pregnant woman?  (Probe: ask for the problems which are not mentioned spontaneously) | Severe bleeding ------------------------------------- 01  Severe headache ------------------------------------- 02  High fever ------------------------------------- 03  Loss of consciousness ----------------------------------- 04  Labor lasting >12 hours ------------------------------- 05  Placenta not delivered 30 minutes after baby ........06  Others specify _________________________97  Don’t know ......................................98 | | | | DK→306 |
| 304 | In your opinion, what are some serious health problems that can occur during the first 2 days after birth that could endanger the life of the woman?  PROBE: Any others? | Severe bleeding................................01  Severe headache ..............................02  Swollen hands/face........................03  High fever ........................................04  Malodorous vaginal discharge 05  Loss of consciousness ..................06  Other ,Specify ________________ 97  Don’t know...........................................98 | | | | DK→308 |
| 305 | Can a woman die from any of these problems? | Yes—1 No------2 Don't know--------98 | | | |  |
| 306 | Have you ever heard the term “birth preparedness”? | Yes ............................................................ 1  No.............................................................. 2 | | | |  |
| 307 | What are some things a woman can do to prepare for birth? | Identify mode of transport. .......01  Save money....................................02  Identify blood donor ...................03  Identify skilled provider..............04  Others,Specify______________97 | | | |  |
| 308 | Does the health system/ community provide services to assist women in preparing for birth?  For instance, are there:  Transportation services for birthing women? Fee free birth services/ways to get money to help families pay for birth?  Ways to get blood donated during pregnancy or complications? Any other services? |  | **YES** | **NO** | **DK** |  |
|  |  | Transport | 1 | 2 | 8 |  |
|  |  | Fee free service/money | 1 | 2 | 8 |  |
|  |  | Blood | 1 | 2 | 8 |  |
|  |  | Other ____________________  (specify) | 1 | 2 | 8 |  |
| 309 | What do you think the advantages of pregnancy and delivery related services? |  | **YES** | **NO** | **DK** |  |
|  |  | For anticipating problems | 1 | 2 | 8 |  |
|  |  | For early detection of health Problems | 1 | 2 | 8 |  |
|  |  | For appropriate management of health problems | 1 | 2 | 8 |  |
|  |  | For better health care to the women | 1 | 2 | 8 |  |
|  |  | For better care to the newborn | 1 | 2 | 8 |  |
|  |  | Others Specify________________ 97 | 1 | 2 | 8 |  |

**Section 4. Partner’s role towards birth planning**

| ***No*** | ***Questions*** | ***Alternative /choice of response*** | ***Skip*** |
| --- | --- | --- | --- |
| 401 | A woman should plan ahead of time where she will give birth to her baby | Strongly Agree, Agree Disagree, Strongly disagree Don’t know  1 2 3 4 8 |  |
| 402 | A woman should plan ahead of time how she will get to the place where she will give birth | Strongly Agree, Agree Disagree, Strongly disagree Don’t know  1 2 3 4 8 |  |
| 403 | When women do not go to a health facility to give birth, it is mainly because it is too expensive. | Strongly Agree, Agree Disagree, Strongly disagree Don’t know  1 2 3 4 8 |  |
| 404 | When women do not go to a health facility to give birth, it is mainly because it is too difficult to get there | Strongly Agree, Agree Disagree, Strongly disagree Don’t know  1 2 3 4 8 |  |
| 405 | When women do not go to a health facility to give birth, it is mainly because the staff there do not treat women respectfully | Strongly Agree, Agree Disagree, Strongly disagree Don’t know  1 2 3 4 8 |  |
| 406 | It is not necessary for a husband/ partner to accompany his wife to antenatal care visits or when she is giving birth | Strongly Agree, Agree Disagree, Strongly disagree Don’t know  1 2 3 4 8 |  |
| 407 | Giving birth is mostly a woman’s matter  Husbands/partners have little to contribute | Strongly Agree, Agree Disagree, Strongly disagree Don’t know  1 2 3 4 8 |  |
| 408 | When women do not go to a health facility to give birth, it is mainly because the staff there do not know what kind of care a woman needs during pregnancy, childbirth and in case of complications | Strongly Agree, Agree Disagree, Strongly disagree Don’t know  1 2 3 4 8 |  |

**Section 5. Perceptions of local facilities**

| ***No*** | ***Questions*** | ***Alternative /choice of response*** | ***Skip*** |
| --- | --- | --- | --- |
| 501 | Do you know of a place where a woman can go to give birth to a baby with assistance from a skilled provider?  IF YES: Where is that?__________  (name of health facility | Home ...............1  Health facility...............2  Other _________________________ 97  (specify)  Does not know place ....................98 | 1→ Sec. 6  98→ Sec. 6 |
| 502 | How would a woman go to this health facility?  PROBE: What type of transportation would she mainly use to get to the health facility? | Ambulance .......................................01  Private car........................................02  Taxi/bus ...........................................03  Cart ....................................................04  On foot................................................05  Other , specify ______________ 97 |  |
| 503 | In general, how long would it take to reach this health facility? | 1.....Hours _______________  2 ....Minutes_______________ | < 2 hours, record in minutes |
| 504 | In your opinion, how are the services in this facility? Would you say they are excellent, good, average, or poor? | Excellent Good Average Poor Don’t know  1 2 3 4 98 |  |
| 505 | Can you tell me why you have ranked the services as [CHECK 504] __________?  PROBE: What else?  RECORD ALL RESPONSES. | Provider always there ...................01  Facility always open ..................02  Facility always has necessary medicines.......03  Not a long wait..........................04  Staff treat women with respect....05  Often provider not there..............06  Unable to perform cultural ceremonies ....07  Often facility is closed...............08  Facility does not have necessary medicines....09  Long wait to be seen ...............10  Staff treat women poorly........11  Other, specify ____________ 97 |  |

**Section 6. Personal experience related to last pregnancy**

| ***No*** | ***Questions*** | ***Alternative /choice of response*** | | | | ***Skip*** |
| --- | --- | --- | --- | --- | --- | --- |
| 601 | Check 106: has had a live birth  Or stillbirth in the last 24 Months? | Yes ............................................................ 1  No.............................................................. 2 | | | | Yes →604  No → Sec. 8 |
| 602 | Any visit to health facility at last pregnancy? | Yes ............................................................ 1  No.............................................................. 2 | | | | No →610 |
| 603 | What was your reason for visiting Health Facility? | For ANC ...................................................01  For birth --------------------------------02  Other, specifay_________________97 | | | | 02 → Sec. 7  97→ Sec. 8 |
| 604 | Did you see anyone for antenatal care during this pregnancy? | Yes ............................................................ 1  No.............................................................. 2 | | | | No →611 |
| 605 | How many times in total did you receive antenatal care during your pregnancy? | 1visit--------1 2-3visits-----2  4 visit------3 4+visits----4 | | | |  |
| 606 | How many months pregnant were you when you first received antenatal care for this pregnancy?) | Months..............................................  Don’t know/don’t remember ............98 | | | |  |
| 607 | Whom did you first see for a checkup on this pregnancy?  Anyone else? Probe for the type of person and record all persons seen. | Doctor.................................................01  Midwife/ Nurse................................02  HEW...................................................03  TBA----------------------------------- 04  Other, specifay_________________97 | | | |  |
| 608 | How many months pregnant were you when you last received antenatal care for this pregnancy? | Months..............................................  Don’t know/don’t remember ............98 | | | |  |
| 609 | During this pregnancy, did a health worker advise you about any of the following at least once?  Danger signs of serious health problems during pregnancy, childbirth, or soon after?  Where to go if you had danger signs of serious health problems?  Where you give birth to your baby?  Arrangements for transportation?  Arrangements for Money ?  Arrangements for a blood donor?  Arrangements for a skilled provider? | Danger signs  Where to go  Where to Give birth  Transport  Fee free services/money  Blood donor  Skilled provider | Yes | NO | DK |  |
|  |  |  | 1  1  1  1  1  1  1 | 0  0  0  0  0  0  0 | 8  8  8  8  8  8  8 |  |
| 610 | Did you speak with anyone outside of a health facility about where to go if you had danger signs of serious health problems, where you should give birth to your baby and arrangements for transport, money, a blood donor & a skilled provider to deliver your child??  IF YES: Whom did you speak with? | Husband........................................ 01  My family & relatives................ 02  Friend/neighbor .........................03  HEW ..............................................04  TBA ................................................05 | Yes | NO | DK |  |
|  |  |  | 1  1  1  1  1 | 0  0  0  0  0 | 8  8  8  8  8 |  |
| 611 | Why did you not see anyone for antenatal care? (CIRCLE ALL RESPONSES GIVEN.) | Did not know where to go.........01  Health facility too far.................02  Too expensive ......................... 03  No one was there to accompany 04  No good service....................... 05  Other,specifay_____________97 |  |  |  |  |

**Section 7. Personal experience related to last birth**

| ***No*** | ***Questions*** | ***Alternative /choice of response*** | ***Skip*** |
| --- | --- | --- | --- |
| 701 | Where did you give birth to your last child?  ________________________________  (NAME OF PLACE) | Home ........................ 1  Health facility ........................ 2  Other, specifay__________97 |  |
| 702 | Prior to this birth, did you or your family make any arrangements for the birth of this child? | Yes—1  No-----2 | No → 704 |
| 703 | Which arrangements did you or your family make for the birth of this child?  (CIRCLE ALL RESPONSES GIVEN.)  THEN PROBE: Did you [ANY  REMAINING ARRANGEMENTS]? | Identify  Transport ..............01  Save money ..................02  Identify blood donor .......03  Skilled provider ..........04  Other, specify___________________ 97 |  |
| 704 | Who made the final decision about where you would give birth? ( Women’s autonomy) | Just me ............................................ 01  My husband/ partner................... 02  My family & relatives.....................03  Religious/cultural leaders............. 04  Friend/neighbor ............................. 05  Health professionals ......................06  Other, specify__________________ 97 |  |
| 705 | How long did it take to make the decision about whether or not to go somewhere for assistance? | 1.....Hours _______________  2 ....Minutes_______________ |  |
| 706 | CHECK 701: GAVE BIRTH IN FACILITY? | Yes—1  No-----2 | No → 716 |
| 707 | Can you tell me the three top reasons why you gave birth in a health facility rather than elsewhere? PROBE: What else? | 1 ___________________________________  2 ___________________________________  3 ___________________________________ |  |
| 708 | How did you go to the health facility?  PROBE: What type of transportation did you mainly use to get to the health facility? | Ambulance .......................................01  Private car........................................02  Taxi/bus ...........................................03  Cart ....................................................04  On foot................................................05  Other , specify ______________ 97 |  |
| 709 | Who accompanied you to the place where you gave birth?  PROBE FOR THE PERSON(S) ACCOMPANYING AND RECORD ALL PERSONS. | No one....................................................... 01  My husband/ partner............................. 02  My family & relatives. ........................ 03  Friend/neighbor ................................... 04  Health professionals ......................... 05  TBA ...........................................................06  Other, specify__________________ 97 |  |
| 710 | How long did it take to find transport once a decision was made to seek care? | 1.....Hours _______________  2 ....Minutes______________ | < 2 hours, record in minutes |
| 711 | How long did it take to reach the health facility? | 1.....Hours _______________  2 ....Minutes______________ | < 2 hours, record in minutes |
| 712 | How long after reaching the health facility did it take for you to get services from the health personnel? | 1.....Hours _______________  2 ....Minutes_______________  3..... Immediately  Don’t know ............................................. 98 | < 2 hours, record in minutes |
| 713 | In your opinion, how were the services in this facility? Would you say they were excellent, good, average or poor? | Excellent Good Average Poor Don’t know  1 2 3 4 98 |  |
| 714 | Can you tell me why you have ranked the services as __________? [CHECK 713]  PROBE: What else? | Provider always there ...................01  Facility always open ..................02  Facility always has necessary medicines.......03  Not a long wait..........................04  Staff treat women with respect....05  Often provider not there..............06  Unable to perform cultural ceremonies ....07  Often facility is closed...............08  Facility does not have necessary medicines....09  Long wait to be seen ...............10  Staff treat women poorly........11  Other, specify ____________ 97 |  |
| 715 | CHECK 701: GAVE BIRTH IN FACILITY? | Yes—1  No-----2 | Yes → 717 |
| 716 | Can you tell me the three top reasons why you did not give birth in a health facility?  PROBE: What else? | Resp. Didn’t think necessary.......... 01  Facility too far.................................... 02  No transport........................................ 03  Too expensive......................................04  Services are poor............................... 05  Unable to perform cultural ceremonies ...06  No time to go ......................................... 07  Other, specify ____________ 97 |  |
| 717 | Who assisted with the birth?  Anyone else?  PROBE FOR THE TYPE OF PERSON AND RECORD ALL PERSONS ASSISTING. | Doctor.................................................01  Midwife/ Nurse................................02  HEW...................................................03  TBA----------------------------------- 04  Other, specifay_________________97 |  |
| 718 | During labor and birth, did you experience any serious health problems related to birth? | Yes—1  No-----2 | No →Sec. 8 |
| 719 | Can you mention what problem/s did you experience? | ___________________________________  ___________________________________ |  |
| 720 | Where were you when you developed  this /these problem/s? | Home ........................ 1  Health facility ........................ 2 | → 722 |
| 721 | Did you seek assistance for this problem? | Yes—1  No-----2 |  |
| 722 | Whom did you see for assistance for this health problem?  Anyone else?  Probe for the type of person and record all persons seen | Doctor.................................................01  Midwife/ Nurse................................02  HEW...................................................03  TBA----------------------------------- 04  Other, specifay_________________97 |  |

**Section 8. Participation in community interventions**

| ***No*** | ***Questions*** | ***Alternative /choice of response*** | ***Skip*** |
| --- | --- | --- | --- |
| 801 | Have you participated in any community activities related to birth preparedness in the past six months? | Yes.................................................................... 01  No ..................................................................... 02  Don’t remember.......................................... 98 | No →  End  interview |
| 802 | Through which activities did you hear  about birth preparedness? (CIRCLE  ALL RESPONSES GIVEN.)  PROBE: Any other sources? | Street drama .............................................. 01  Community meetings ................................. 02  Women’s groups ....................................... 03  Literacy groups ........................................ 04  Other, specify____________________ 97 |  |
| 803 | What messages related to birth preparedness that you learned about through these activities?  PROBE: Any other messages? | Identify  Transport ..............01  Save money ..................02  Identify blood donor .......03  Skilled provider ..........04  Other, specify_____________97 |  |
| 804 | Did you do anything or take any action related to birth preparedness after learning about these topics? | Yes.................................................................... 01  No ..................................................................... 02  Don’t remember.......................................... 98 |  |
| 805 | What action(s) did you take? (CIRCLE  ALL RESPONSES GIVEN.)  PROBE: Anything else? | Arrange transportation ...................... 01  Arrange money........................................... 02  Arrange blood donor............................. 03  Arrange skilled provider..................... 04  Other, specify__________________ 97 |  |

THANK THE RESPONDENT
